# Regulation of enhanced cerebrovascular expression of proinflammatory mediators in experimental subarachnoid hemorrhage via the mitogen-activated protein kinase kinase/extracellular signal-regulated kinase pathway

**DOI:** 10.1186/1742-2094-9-274

**Published:** 2012-12-21

**Authors:** Aida Maddahi, Gro Povlsen, Lars Edvinsson

**Affiliations:** 1 Department of Clinical Sciences, Division of Experimental Vascular Research, Lund University, Lund, Sweden; 2 Department of Clinical Experimental Research, Glostrup University Hospital, Glostrup, Denmark

**Keywords:** Subarachnoid hemorrhage, TNFα, IL-1β, IL-6, MMP-9, Phosphorylated ERK1/2

## Abstract

**Background:**

Subarachnoid hemorrhage (SAH) is associated with high morbidity and mortality. It is suggested that the associated inflammation is mediated through activation of the mitogen-activated protein kinase (MAPK) pathway which plays a crucial role in the pathogenesis of delayed cerebral ischemia after SAH. The aim of this study was first to investigate the timecourse of altered expression of proinflammatory cytokines and matrix metalloproteinase in the cerebral arteries walls following SAH. Secondly, we investigated whether administration of a specific mitogen-activated protein kinase kinase (MEK)1/2 inhibitor, U0126, given at 6 h after SAH prevents activation of the MEK/extracellular signal-regulated kinase 1/2 pathway and the upregulation of cerebrovascular inflammatory mediators and improves neurological function.

**Methods:**

SAH was induced in rats by injection of 250 μl of autologous blood into basal cisterns. U0126 was given intracisternally using two treatment regimens: (A) treatments at 6, 12, 24 and 36 h after SAH and experiments terminated at 48 h after SAH, or (B) treatments at 6, 12, and 24 h after SAH and terminated at 72 h after SAH. Cerebral arteries were harvested and interleukin (IL)-6, IL-1β, tumor necrosis factor α (TNF)α, matrix metalloproteinase (MMP)-9 and phosphorylated ERK1/2 (pERK1/2) levels investigated by immunohistochemistry. Early activation of pERK1/2 was measured by western blot. Functional neurological outcome after SAH was also analyzed.

**Results:**

Expression levels of IL-1β, IL-6, MMP-9 and pERK1/2 proteins were elevated over time with an early increase at around 6 h and a late peak at 48 to 72 h post-SAH in cerebral arteries. Enhanced expression of TNFα in cerebral arteries started at 24 h and increased until 96 h. In addition, SAH induced sensorimotor and spontaneous behavior deficits in the animals. Treatment with U0126 starting at 6 h after SAH prevented activation of MEK-ERK1/2 signaling. Further, U0126 significantly decreased the upregulation of inflammation proteins at 48 and 72 h following SAH and improved neurological function. We found no differences between treatment regimens A and B.

**Conclusions:**

These results show that SAH induces early activation of the MEK-ERK1/2 pathway in cerebral artery walls, which is associated with upregulation of proinflammatory cytokines and MMP-9. Inhibition of the MEK-ERK1/2 pathway by U0126 starting at 6 h post-SAH prevented upregulation of cytokines and MMP-9 in cerebral vessels, and improved neurological outcome.

## Background

Subarachnoid hemorrhage (SAH) (caused by rupture of a cerebral arterial aneurysm, an arterial-venous malformation, or a head trauma) is associated with high morbidity and mortality. It may be associated with an acute rise of intracranial pressure (ICP), a reduction in cerebral blood flow (CBF), rebleeding, vasospasm and delayed cerebral ischemia [1]. Clinical and experimental studies have shown a marked inflammatory response in the brain and subarachnoid space after SAH [2-5], and in SAH patients a systemic inflammatory response has been demonstrated with circulating immune complexes, complement activation, increased levels of cytokines and endothelial adhesion molecules [6-12]. It is generally believed that the inflammation response has a crucial role in tissue repair in the brain but it can also be detrimental to the affected tissue, and it has been suggested to play a key role in progression of brain damage inferred in the acute stage of SAH as well as in the later development of cerebral vasospasms and delayed cerebral ischemia.

It has been proposed that the primary mechanism triggering inflammation after SAH is oxyhemoglobin from extravasated blood [13]. Oxyhemoglobin triggers generation of free radicals, known to be powerful initiators of vascular damage and inflammation. The inflammatory response after SAH involves activation of platelets around spastic arteries, activation and accumulation of macrophages, leukocytes and monocytes in the subarachnoid space and the intima of cerebral arteries, and is associated with increased levels of inflammatory mediators [14,15].

Inflammatory mediators involved after SAH include the proinflammatory cytokines interleukin (IL)-1β, IL-6 and tumor necrosis factor α (TNFα), all of which have been demonstrated in increased amounts in the cerebrospinal fluid (CSF) or blood from SAH patients [6,16-18]. Indeed, many clinical signs associated with brain injury after SAH such as neutrophilia, pyrexia and cerebral edema are thought to be caused by cytokine activity [19]. Another family of inflammatory mediators that appear to be of importance after SAH are the matrix metalloproteinases (MMPs), primarily MMP-9 [20-22]. Clinical studies have shown increased MMP-9 levels in brain, CSF and peripheral blood after SAH [23-25]. MMP-9 degrades tight junction proteins and components of the basal lamina, which results in disruption of the blood-brain barrier (BBB). This leads to the formation of brain edema, which contributes to the neuroinflammatory response, brain damage and poor outcome after SAH [26,27].

Whereas earlier studies have focused on cytokine and MMP expression in CSF, blood and/or brain tissue after experimental and/or clinical SAH, we have studied the role of cytokines and MMPs in the walls of cerebral arteries after SAH. Using microarrays, we have demonstrated upregulation of IL-6, IL-1β, TNFα and MMP-9 mRNA levels in the smooth muscle layer of cerebral arteries after experimental SAH [28]. At 48 h post-SAH there is a reduction in CBF, enhanced expression of cytokines and MMP-9, and activation of the extracellular signal-regulated kinase 1 and 2 (ERK1/2) pathway both in cerebral arteries and microvessels [29]. Furthermore, specific blockade of the mitogen-activated protein kinase kinase (MEK)-ERK1/2 pathway by inhibition of the upstream Raf protein prevented the reduction in CBF and abolished the upregulation of cytokines and MMP-9 [29], suggesting a crucial role of this pathway in the cerebrovascular inflammatory response, and delayed cerebral ischemia after SAH. However, there is limited information on the timecourse of the increase in expression of cytokines and MMP-9 in brain vessels following SAH, and it is also somewhat unclear whether this upregulation is specifically located to brain blood vessels or cerebral parenchyma. Moreover, the critical time period for involvement of the MEK-ERK1/2 signaling pathway as a mediator of cerebrovascular inflammation has not been explored. Therefore, the present study was designed to examine the timecourse of upregulation of cerebrovascular proinflammatory cytokines and MMP-9 over 96 h in a rat SAH model that approximates late cerebral ischemia [30,31]. In addition, we address the hypothesis that the cerebrovascular cytokine response after SAH is associated with early activation of the MEK-ERK1/2 pathway, and that inhibition of this pathway by a specific MEK1/2 inhibitor, U0126, given only in the time window from 6 to 24 h post-SAH can modify the cerebrovascular inflammatory response as well as the neurological outcome several days post-SAH.

## Materials and methods

### Animals

All animal procedures were carried out strictly within national laws and guidelines and were approved by the Danish Animal Experimentation Inspectorate (license no. 2011/561-2025) and the Ethical Committee for Laboratory Animal Experiments at the University of Lund (license no: M8-09).

### Rat subarachnoid hemorrhage model

SAH was induced as described in detail previously [31]. Male Sprague-Dawley rats (350 to 400 g) were anesthetized using 3.5% isoflurane (Abbott Laboratories, IL, USA) in atmospheric air N_2_O/O_2_ (70:30). Rats were orally intubated and kept on artificial ventilation with inhalation of 1% to 2% isoflurane in N_2_O/O_2_ (70:30) during the surgical procedure. Respiration was monitored by regularly withdrawing blood samples to a blood gas analyzer (Radiometer, Copenhagen, Denmark). A temperature probe was rectally inserted to record the body temperature, which was maintained at 37°C via a heating pad. Intracranial pressure (ICP) was measured via a catheter inserted into the basal cistern via a hole in the atlanto-occipital membrane. The catheter was connected to a pressure transducer and the signal was recorded in the software LabChart via a PowerLab (both from AD Instruments, Oxford, UK). Mean arterial blood pressure (MABP) was measured via a tail artery catheter, likewise connected to a pressure transducer and recorded in LabChart. Cortical cerebral blood flow (CBF) was measured with a laser-Doppler fiber-optic probe placed directly on the dura mater on the surface of the brain via a hole drilled through the skull, 4 mm anterior from the bregma and 3 mm to the right of the midline without perforation of the dura. ICP, MABP and CBF were all measured in real time with recordings commencing app. 30 minutes before SAH and continuing until 1 h after the SAH.

A 27 G blunt cannula with a side hole facing right was stereotactically placed 6.5 mm anterior to the bregma in the midline at an angle of 30º to the vertical plane, placing the tip of the needle just in front of the chiasma opticum. After 30 minutes of equilibration, during which the level of anesthesia was adjusted to obtain a MABP of 80 to 100 mmHg, 250 μl of blood was withdrawn from the tail catheter and injected manually into the prechiasmatic cistern at a pressure equal to the MABP. Subsequently, rats were maintained under anesthesia for another 60 minutes in order to allow the animal to recover. The ICP catheter was cut and sealed with a removable plug 2 cm from the tip. The tail catheter, the needle and the laser-Doppler probe were removed and incisions closed. Rats were revitalized and extubated. At the end of the operation and every 24 h until termination, rats received a subcutaneous injection of carprofen (4 mg/kg), (Pfizer, Ballerup, Denmark) and 15 ml of isotonic saline subcutaneous for hydration. Sham-operated rats went through the same procedure, with the exception that no blood was injected intracisternally.

### U0126 and vehicle treatment groups

A total of 83 rats were operated on for this study. Table 1 summarizes the distribution of the rats into experimental groups. In groups including treated animals, animals were randomly selected for treatment with either U0126 or vehicle. U0126 was given as 0.05 ml/kg body weight of a 10^-5^ M solution of U0126 ethanolate (Sigma-Aldrich, St Louis, MO, USA) in isotonic saline with 0.1% dimethylsulfoxide (DMSO), yielding a final dose of 0.22 μg U0126/kg body weight. Vehicle was 0.1% DMSO in isotonic saline. Treatment was administered intracisternally through the ICP catheter placed with the tip in the basal cistern. 

**Table 1:** Summary of experimental groups

**Experimental group**	**N**	**Time after SAH**								
		**2 min**	**1 h**	**6 h**	**12 h**	**24 h**	**36 h**	**48 h**	**72 h**	**96 h**
Immunohistochemistry: temporal profile of proinflammatory mediators, pERK1/2 and effect of U0126										
0 h (2 min SAH)	3	X								
1 h SAH	3		X							
6 h SAH	3			X						
12 h SAH	3				X					
24 h SAH	3					X				
48 h sham	6							X		
48 h SAH + vehicle	5			V	V	V	V	X		
48 h SAH + U0126	6			U	U	U	U	X		
72 h sham	5								X	
72 h SAH + vehicle	6			V	V	V			X	
72 h SAH + U0126	6			U	U	U			X	
96 h sham	7									X
96 h SAH	6									X
Western blots: ERK activation at early timepoints after SAH										
1 h sham	3		X							
1 h SAH	3		X							
6 h sham	3			X						
6 h SAH	3			X						
24 h sham	3					X				
24 h SAH + vehicle	3			V	V	X				
24 h SAH + U0126	3			U	U	X				

X indicates termination of all rats in each group. V indicates treatment with vehicle (0.01% dimethylsulfoxide) and U indicates treatment with U0126.

ERK = extracellular signal-regulated kinase; N = no.of rats in each group; SAH = subarachnoid hemorrhage.

In order to assess whether MEK-ERK1/2 activity is continuously involved in the vascular inflammatory response throughout the first 3 days post-SAH or whether this pathway acts as an acute ‘switch-on’ mechanism for the upregulation of inflammatory mediators, we compared the effect of U0126 given under two different treatment regimens: (A) continuously, 6, 12, 24 and 36 h post-SAH with rats killed at 48 h post- SAH; and (B) acutely, 6, 12 and 24 h followed by a 2-day period without any treatment with rats killed at 72 h post-SAH.

### Neurological function

Functional neurological outcome after SAH was analyzed by two tests: (1) rotating pole test, and (2) standardized observations of spontaneous activity.

All neurological evaluations (rotating pole test and spontaneous behavior observations) were performed by personnel blinded with regard to the experimental groups of the animals. Tests and observations were performed in the morning to minimize diurnal rhythm variation and in a silent room with as few potentially distracting visual elements as possible.

#### Rotating pole test

Gross sensorimotor function (integration and coordination of movements as well as balance) was evaluated by the ability of the rats to traverse a rotating pole, which was either steady or rotating at different speeds (3 or 10 rpm) [32]. At one end of the pole (45 mm in diameter and 150 cm in length), a cage was placed with an entrance hole facing the pole. The floor of the cage was covered with bedding material from the home cage of the rat being tested, thus serving as a positive reinforcement for the rat to traverse the pole when placed at the end opposite to the cage. The performance of the rat was scored according to the following definitions: score 1, the animal is unable to balance on the pole and falls off immediatelyscore 2, the animal balances on the pole but has severe difficulties crossing the pole and moves less than 30 cm; score 3, the animal embraces the pole with the paws and does not reach the end of the pole but manages to move more than 30 cm; score 4, the animal traverses the pole but embraces the pole with the paws and/or jumps with the hind legs; score 5, the animal traverses the pole with normal posture but with more than three to four foot slips; score 6, the animal traverses the pole perfectly with less than three to four foot slips.

#### Spontaneous behavior observations

Spontaneous activity of the rats was quantified by placing the rats individually in a test cage with fresh bedding and nesting material for 20 minutes. An observer equipped with a timer recorded all time intervals spend moving around in the cage (locomotion), sitting or lying in the same place (no movement), rearing, grooming or eating.

### Harvest of cerebral arteries

At 0, 1, 6, 12, 24, 48, 72 or 96 h after SAH induction or sham surgery, rats were anaesthetized using CO_2_ and decapitated. The brains were quickly removed and chilled in ice-cold bicarbonate buffer solution of the following composition (mM): 119 NaCl, 15 NaHCO_3_, 4.6 KCl, 1.2 MgCl_2_, 1.2 NaH_2_PO_4_, 1.5 CaCl_2_ and 5.6 glucose. Under a dissection microscope, the middle cerebral arteries (MCAs) and the basilar artery (BA) were carefully dissected either as isolated structures free from the brain tissue for westerns or together with surrounding brain tissue (a square of approximately 0.3 cm^2^ thickness around each artery) for immunohistochemistry and snap frozen at −80°C.

### Histochemistry

#### Hematoxylin and eosin staining

Hematoxylin and eosin (H&E) staining is a routine method in histology that shows cytoplasm stained red and cell nuclei stained blue or purple. Here, both MCA and BA sections from fresh, 0 h, 1 h, 6 h, 12 h and 24 h post-SAH rats were fixed for 10 minutes in ice-cold acetone and then rehydrated in phosphate buffered saline (PBS) containing 0.25% Triton X-100 for 15 minutes. Subsequently, they were stained with H&E to evaluate the effects of SAH on the morphology of cerebral arteries and surrounding brain tissue. Briefly, sections were incubated for 4 minutes in hematoxylin, washed with running water and rinsed in distilled water. Thereafter, the sections were immersed in eosin for 1 minute, dehydrated in ethanol and mounted with Pertex mounting medium (Histolab Products AB, Gothenburg, Sweden).

### Immunohistochemistry

Cerebral arteries (MCA and BA) with surrounding brain tissue from sham, SAH, SAH + vehicle and SAH + U0126 rats were dissected out, embedded in Tissue TEK (Gibo, Invitrogen A/S, Taastrup, Denmark), and frozen on dry ice. Thereafter, they were sectioned into 10-μm thick slices in a cryostat (Microm HM500M; Thermo Scientific, Walldorf, Germany). Three sections were collected and placed on each microscope slide (Menzel, Branuschweig, Germany). Subsequently, sections were fixed for 10 minutes in ice-cold acetone and then rehydrated in phosphate-buffered saline (PBS) containing 0.25% Triton X-100 for 15 minutes. The tissue was then permeabilized and blocked for 1 h in blocking solution containing PBS, 0.25% Triton X-100, 1% bovine serum albumin (BSA), and 5% normal donkey serum. The sections were incubated overnight at 4°C with the following primary antibodies: rabbit anti-rat MMP-9 (Abcam, ab7299), rabbit anti-rat IL-6 (Abcam, ab6672), rabbit anti-rat IL-1β (Abcam, ab9787) diluted 1:400, rabbit polyclonal to TNFα (Abcam, ab15563) diluted 1:500, and rabbit anti-phospho-ERK1/2 (Cell Signaling Technology #4376, Danvers, MI, USA) diluted 1:50. All dilutions were made in PBS containing 0.25% Triton X-100, 1% BSA, and 2% normal donkey serum. Sections were subsequently washed with PBS and incubated with secondary antibody for 1 h at room temperature. The secondary antibody used was donkey anti-rabbit Dy-Light 488 conjugate (Jackson ImmunoResearch Europe Ltd., Newmarket, UK) diluted 1:200 in PBS containing 0.25% Triton X-100 and 1% BSA. The sections were subsequently washed with PBS and mounted with anti-fading vectashield mounting medium (Vector Laboratories Inc., Burlingame, CA USA). Immunoreactivity was visualized using an epifluorescence microscope (Nikon 80i; Tokyo, Japan) at the appropriate wavelengths and photographed with an attached Nikon DS-2Mv camera. The same procedure was used for the negative controls except that either the primary antibody or the secondary antibody was omitted to verify that there was no autofluorescence or unspecific labeling.

### Double immunohistochemistry

Double immunohistochemistry was performed for TNFα and glial fibrillary acidic protein (GFAP), an astrocyte/glial cell marker. In addition to the antibodies described above, we used mouse anti-GFAP (G3893; Sigma) diluted 1:600 in PBS containing 0.3% Triton X-100, 1% BSA, and 2% normal donkey serum. The secondary antibodies were donkey anti-rabbit Dy-Light 488 and donkey anti-mouse antibody Dy-Light 549 (both from Jackson ImmunoResearch, Europe Ltd. Newmarket, UK)) diluted 1:200 in PBS containing 3% Triton X-100 and 1% BSA. The antibodies were detected at the appropriate wavelengths using a confocal microscope (Nikon, EZ-cl, Dusseldorf, Germany).

### Image analysis

Fluorescence intensity was measured using ImageJ software ( http://rsb.info.nih.gov/ij/). Measurements were made in four areas (located on the clock at 0, 3, 6, and 9 h) from three vessel sections of each vessel sample. Investigator was blinded to the treatment groups. The fluorescence intensity of each group is given as the percentage fluorescence of the SAH + vehicle group compared to the sham and/or 0 h groups, where the sham or 0 h group was set to 100%, and the mean value for each of the other groups was used for comparison.

### Western blot

The BA and both MCAs were isolated from each rat and pooled into one sample for each rat (n = 3 rats in each group). Arteries were homogenized by sonication on ice for 2 minutes in a modified radioimmunoprecipitation assay (RIPA) buffer (50 mM Tris pH 7.5, 150 mM NaCl, 1 mM ethylenediaminetetra-acetic acid (EDTA), 50 mM β-glycerolphosphate, 1% NP-40, 0.1% deoxycholate, 0.1% SDS, 0.5% Triton X-100) containing phosphatase (Merck, Darmstadt, Germany) and protease inhibitor (Sigma) cocktails. Sonicated tissue lysates were centrifuged at 15,000 rpm at 4°C for 30 minutes, and the supernatants were collected as protein samples. Protein concentrations were determined using standard protein assay reagents (Bio-Rad, Hercules, CA, USA) and 15 to 20 μg total protein in each sample was added LDS sample buffer (Expedeon, Cambridge, UK) and separated on a 4% to 15% RunBlue SDS Precast gel (Expedeon) at 180 V for 60 minutes and then transferred to a polyvinylidene difluoride (PVDF) membrane at 150 V for 70 minutes. Membranes were blocked in blocking buffer (PBS containing 0.1% Tween-20 and 2% ECL Advance blocking agent (GE Healthcare, Chalfont St Giles, UK)) for 1 h at room temperature and then incubated overnight rotating at 4°C in blocking buffer with the primary antibody rabbit anti-rat phospho-ERK1/2 (Cell Signalling Technology) diluted 1:1,000, followed by incubation with ECL horseradish peroxidase (HRP)-conjugated donkey anti-rabbit IgG antibody (GE Healthcare) for 1 h at room temperature. Labeled proteins were developed using Lumigen TMA-6 chemiluminiscence solutions (GE Healthcare). Subsequently, the membranes were extensively washed in PBS + 0.1% Tween-20 and then reprobed with the primary antibody mouse anti-rat total ERK1/2 (Cell Signalling Technology) diluted 1:1,000, at 4°C overnight in blocking buffer followed by incubation with horseradish peroxidase HRP-conjugated goat anti-mouse IgG antibody (Pierce/Thermo Scientific, Rockford, IL, USA) 1:5,000 for 1 h at room temperature and development of labeled proteins. Labeling chemiluminiscence intensities were quantified using the software Image Gauge V4.0 (Fujifilm, Tokyo, Japan).

### Calculation and statistical analyses

Data are expressed as mean ± standard error of the mean (SEM), and n refers to the number of rats. Statistical analyses were performed with two-tailed unpaired Student t tests for comparison between two groups and Kruskal-Wallis non-parametric tests with Dunn’s post hoc tests for comparison of more than two groups, using GraphPad Prism v.5 (GraphPad software, Inc., La Jolla, CA, USA). P values of <0.05 were considered significant.

## Results

### Subarachnoid hemorrhage model

A total of 12% of all SAH rats died within the first 24 h after SAH induction, whereas the mortality for sham-operated rats was 3%. There was no significant difference in the mortality rates between different SAH groups (SAH, SAH + vehicle and SAH + U0126). In all operated rats, mean arterial blood pressure (96.5 ± 9.5 mmHg), partial pCO_2_ (4.7 ± 1.2), partial pO_2_ (17 ± 2.1), pH (7.4 ± 0.6) and body temperature were within normal physiological range during the operation. There was no statistical difference in physiological parameters among the groups. As a result of injecting blood, the cortical blood flow dropped over the right hemisphere to 12.5 ± 10% of resting flow and the ICP increased from 7.2 ± 2 mmHg to 138 ± 38 mmHg. The blood flow and ICP returned to basal values within 1 h after injection. There was no difference in ICP rise and cortical blood flow drop between SAH and U0126 groups. The treatment with U0126 was well tolerated by the animals throughout the 48 h post-SAH period and did not induce any acute change in the above parameters.

### Functional neurological outcome

To characterize the effect of U0126 treatment on neurological deficits after SAH, we employed two tests: (1) a rotating pole test [32], and (2) standardized observations of spontaneous activity. These methods are used in our laboratory to assess functional neurological deficits after experimental SAH [33].

**Figure 1:**
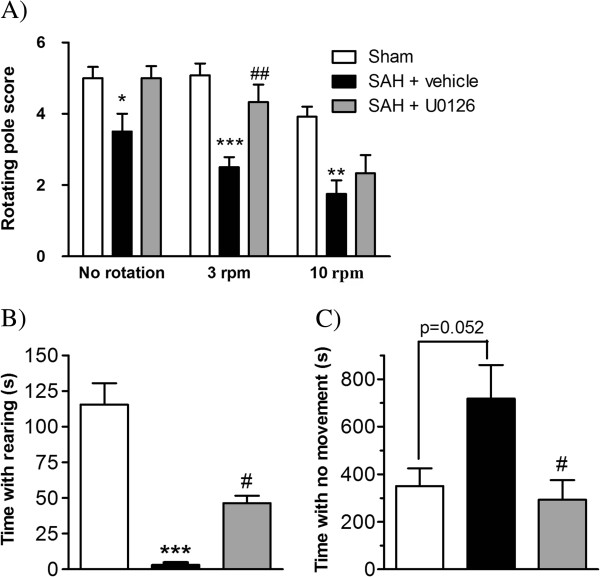
**Neurological function determined by rotating pole test and spontaneous behavior. **(**A**) Rotating pole scores for sham-operated rats, rats with induced subarachnoid hemorrhage (SAH) treated with vehicle and rats induced SAH treated with U0126 at 6 h, 12 h and 24 h after SAH. Scores were obtained at day 3 after SAH with the pole either kept still (no rotation) or rotating at 3 rpm or 10 rpm. (**B**,**C**) Spontaneous activity of rats placed in a clean test cage for a 20 minutes observation period at day 3 after SAH for sham rats and SAH rats treated with vehicle or U0126 at 6 h, 12 h and 24 h after SAH. (**B**) Time spent with rearing. (**C**) Time spent sitting or lying at the same place (no movement). Data are expressed as mean ± SEM, n = 5 in each group. * Indicates significant differences as compared to sham-operated rats, ^#^ indicates significant differences as compared to the SAH group; */^#^*P* <0.05, **/^##^*P* <0.01 and ***P <0.001.

In the rotating pole test, SAH + vehicle-treated rats showed significant deficits compared to sham-operated rats, whereas SAH rats treated with U0126 displayed improved performance (Figure 1A). Similar pattern of changes was observed in the spontaneous behavior of the rats. SAH + vehicle rats tested at 3 days after SAH spend significantly less time rearing than sham-operated rats, and this reduction in rearing time was partly prevented by U0126 treatment (treatment regimen B) (Figure 1B). In addition, time spent with no movement was increased after SAH compared to sham-operated rats and this increase was fully prevented by U0126 treatment (treatment regimen B) (Figure 1C). An earlier study by Larsen and coworkers [33] showed that the effect of U0126 regimen A treatment on performance in the rotating pole test was comparable to the effects shown here using regimen B. 

### Morphological evaluation

**Figure 2:**
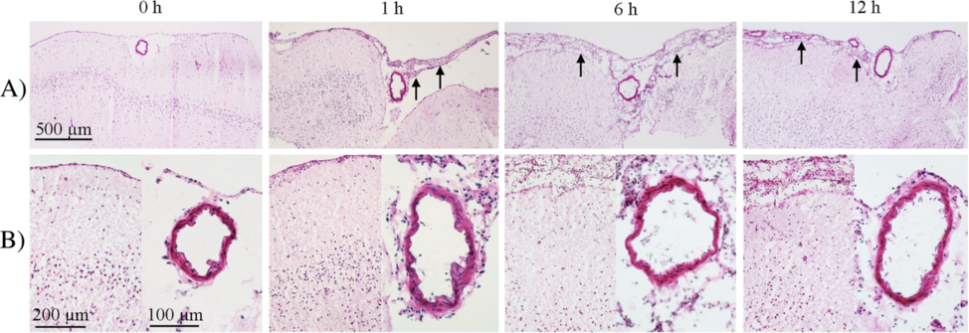
**Hematoxylin and eosin staining of middle cerebral artery **(**MCA**) **sections and surrounding brain tissue following subarachnoid hemorrhage (SAH). **Images represent MCA and surrounding brain tissue (**A**) at 0, 1, 6 and 12 h following SAH. (**B**) High magnification of both MCA and brain tissue. Invading cells (suggesting neutrophils and leucocytes) were observed around MCA and under pia mater at 1 h post-SAH, which increased over time at 6 and 12 h (arrows) as compared to 0 h groups.

The morphology of the MCA, BA and surrounding brain tissue was investigated using routine H&E staining (Figure 2). The staining revealed vessels (MCA and BA) consisting of a clearly visible lamina elastica interna and a medial layer consisting of three to four layers of smooth muscle cells (SMCs). The surrounding brain tissue was often slightly vacuolized, which might be a result of snap freezing. As shown in Figure 2, we found some invading cells surrounding the cerebral arteries, which we suggest might be neutrophils and leukocytes. These cell types have earlier been shown to become activated acutely, extravasate through cerebral arteries and accumulate around the affected vessels following cerebral ischemia [34,35] and subarachnoid hemorrhage [36,37]. The cell invasion was found as early as 1 h post-SAH and increased in the period 6 to 12 h (Figure 2). 

### Proinflammatory mediators in cerebral arteries following SAH

**Figure 3:**
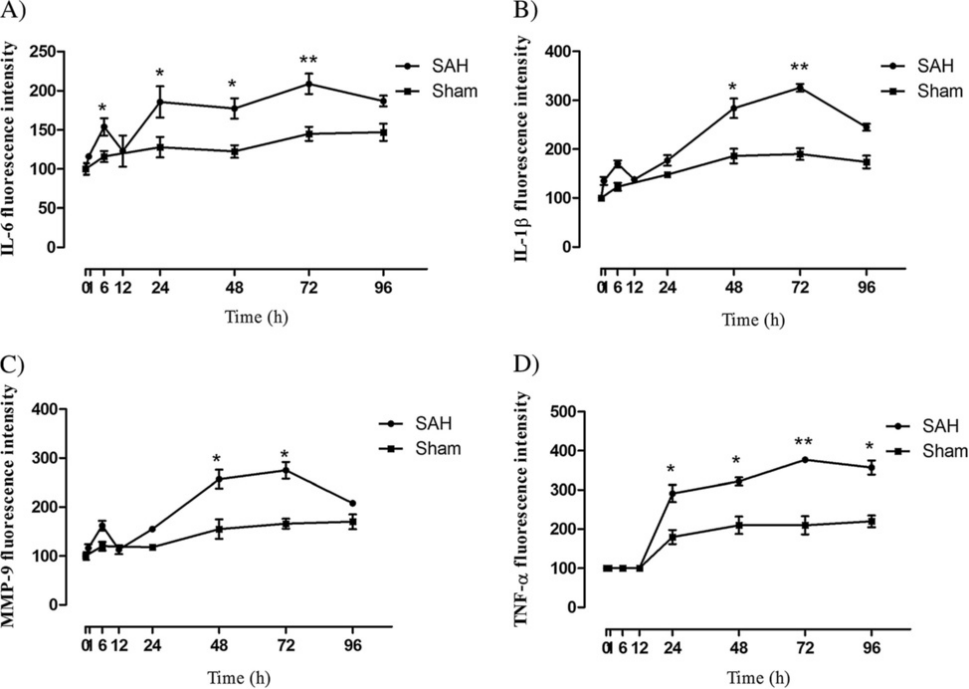
**Fluorescence intensity demonstrating protein levels for interleukin (IL)-6**, **IL-1β**, **matrix metalloproteinase (MMP)-9 and tumor necrosis factor (TNF)α following subarachnoid hemorrhage (SAH) and sham operated animals in the walls of the middle cerebral artery (MCA). **Densitometry analysis showed that there was an early transient increase of IL-6 (**A**) and IL-1β (**B**) protein in the walls of the MCA around 6 h which increased over time during 24 to 96 h with a maximum peak at 72 h after SAH. The MMP-9 protein level showed a slight increase at 6 h and a gradual increase during the period of 24 to 96 h with a maximum increase at 48 to 72 h (**C**). Expression of TNFα protein started during 12 to 24 h, which increased over time until 96 h (**D**). n = 3, * indicates significant differences as compared to sham-operated rats for each timepoint. *P <0.05, **P <0.01.

Protein levels of IL-1β, IL-6, TNFα and MMP-9 in cerebral arteries at 0 to 96 h after SAH and sham operated animals were investigated with immunohistochemistry. A semiquantitative method (fluorescence intensity measurements) was used for analysis of the immunoreactivity. The results showed that IL-1β, IL-6, TNFα, and MMP-9 protein levels in MCA were elevated over time, with maximal expression levels at 72 h after SAH (Figure 3). 

**Figure 4:**
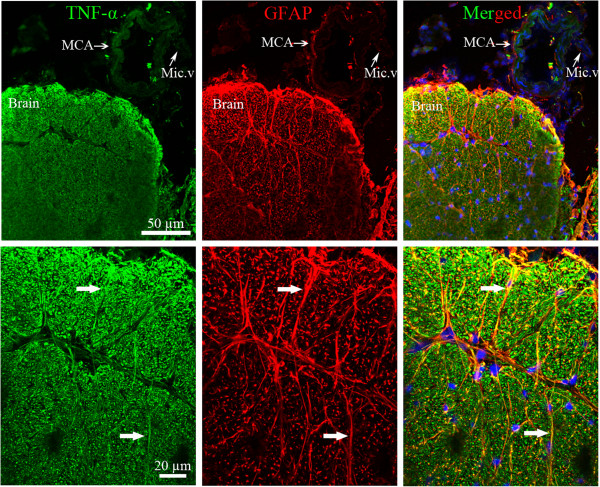
**Double immunofluorescence staining for tumor necrosis factor (TNF)α (green) and glial fibrillary acidic protein (GFAP) (red) in the walls of the basilar artery (BA) and in surrounding brain tissue at 0 to 24 h post**-**subarachnoid hemorrhage (SAH). **There was no expression of TNFα in the walls of the BA at 0 to 24 h post-SAH, but an enhanced TNFα immunoreactivity was seen in the brain tissue. This was colocalized with GFAP (white arrows in merged picture). GFAP expression was observed in astrocytes around BA and in the surrounding brain tissue.

IL-6 (Figure 3A), IL-1β (Figure 3B) and MMP-9 (Figure 3C) immunoreactivities displayed an early peak at 6 h post-SAH followed by a further increase over time until their maximum peaks at 72 h. The expression of TNFα showed a somewhat different distribution compared to the other cytokines analyzed. At 0 to 12 h post-SAH, no TNFα expression was found in the walls of the MCA and BA (Figure 3D), but a strong expression of TNFα was seen in the surrounding brain tissue, mostly in glial cells and neurons (Figure 4). Interestingly, this expression decreased in the brain tissue during 24 to 96 h and instead was increased in the SMCs in cerebral arteries, which was elevated over time (Figure 3D). Thus, the expression of TNFα appears to undergo a shift from expression primarily in brain tissue proper to expression mainly in cerebral vessels around the timepoint 24 h post-SAH. 

### TNFα association with astrocytes in the brain tissue

To address a possible colocalization of TNFα and GFAP in the cerebral arteries and surrounding brain tissue at 0 to 24 h after SAH, double immunofluorescence staining was performed. We detected no GFAP immunoreactivity in the walls of the MCA or BA and microvessels, but observed that there was a rich network of GFAP expression in the brain tissue surrounding cerebral arteries after experimental SAH (Figure 4). Here, we detected an enhanced TNFα immunoreactivity in the brain tissue during 0 to 24 h after SAH, which colocalized with GFAP (Figure 4).

### Phosphorylated ERK1/2 immunoreactivity 2 and 3 days following SAH

**Figure 5:**
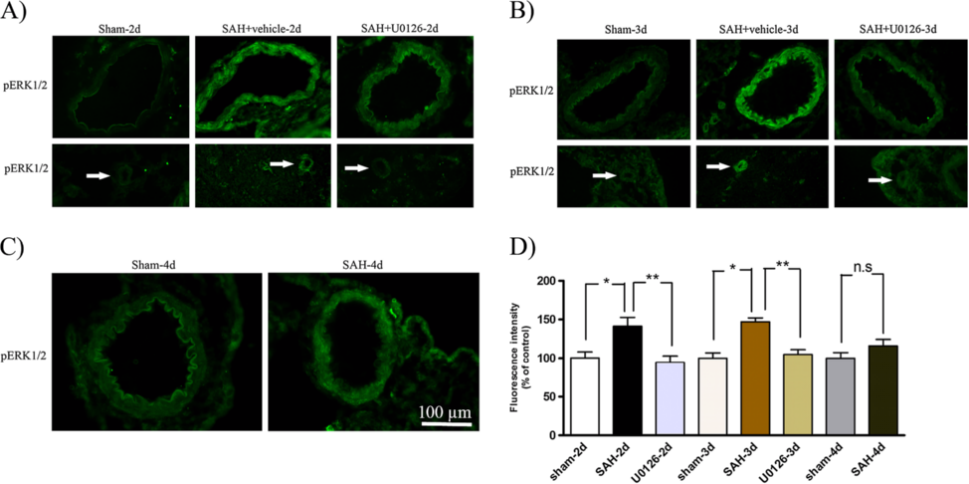
**Phosphorylated extracellular signal**-**regulated kinase (pERK)1/2 immunoreactivity in the walls of the middle cerebral artery (MCA) and microvessels following subarachnoid hemorrhage (SAH). **Images represent (**A**) sham, SAH + vehicle and SAH + U0126, 2 days after SAH; (**B**) sham, SAH + vehicle and SAH + U0126, 3 days after SAH; and (**C**) sham and SAH, 4 days after SAH. There was an increased activation of pERK1/2 in the SMCs of the MCA and microvessels (white arrows) after SAH + vehicle as compared to sham-operated rats at 2 and 3 days. Treatment with U0126 given at 6 h after induced SAH prevented the increased protein expression at 2 and 3 days. (**D**) Bar graph showing semiquantification of fluorescence intensity for pERK1/2 in the MCA. There was a significant increase in the expression of this protein in SAH animals as compared to sham animals at 2 and 3 days post-SAH but not at 4 days. This increase was prevented with U0126 treatment starting at 6 h. Data are presented as the mean percentage relative to sham ± SEM; n = 5. *P <0.05, **P <0.01.

The levels of phosphorylated ERK1/2 (pERK1/2) in the SMCs in the wall of the MCA was significantly increased at 48 and 72 h post-SAH, with maximum at 72 h as compared to time matched sham groups (141 ± 11% and 148 ± 4%,respectively; *P <0.05, **P <0.01). Treatment with U0126 by regimen A (at 6, 12, 24, and 36 h after SAH followed by termination at 48 h after SAH), or regimen B (6, 12, and 24 h after SAH followed by termination at 72 h after SAH) prevented this increased ERK1/2 phosphorylation (95 ± 8%, 110 ± 10%, respectively; *P <0.05). The ERK1/2 immunoreactivity was only slightly increased at 96 h post-SAH compared to sham (116 ± 8%; *P* > 0.05) (Figure 5). In addition, the increase in pERK1/2 immunoreactivity was seen in microvessels at 48 and 72 h post-SAH, but not in brain tissue surrounding the MCA and microvessels. Treatment with U0126 also reduced the microvessel ERK1/2 phosphorylation (Figure 5). 

### ERK1/2 activation at early timepoints after SAH determined by western blot

**Figure 6:**
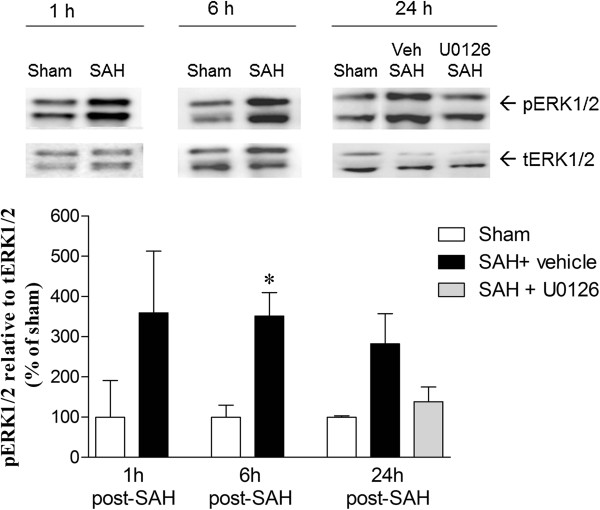
**Western blot showing increased phosphorylated extracellular signal-regulated kinase (pERK)1/2 protein level in cerebral arteries (middle cerebral arteries (MCAs) + the basilar artery (BA)) at 1 h, 6 h and 24 h after subarachnoid hemorrhage (SAH) + vehicle as compared to sham groups. **Treatment with U0126 given at 6 h post-SAH decreased this increase in pERK1/2 at 24 h after SAH. β-Actin was used as a loading control. Data are expressed as mean ± SEM, n = 3. *P <0.05.

To examine the degree of ERK1/2 activation at earlier timepoints following SAH, and to confirm that treatment with U0126 inhibited also this early ERK activity, we performed western blot on cerebral arteries from sham-operated and SAH rats treated with U0126 or vehicle and terminated at 1, 6 and 24 h after surgery. U0126 was administered at 6 h and 12 h after SAH induction, representing the two first treatment times in both regimen A and B. ERK1/2 phosphorylation was increased in the cerebral arteries at 1 h, 6 h and 24 h after SAH, whereas arteries from SAH rats treated with U0126 and terminated at 24 h showed ERK1/2 phosphorylation levels comparable to sham levels (Figure 6). These data show that ERK1/2 is activated in cerebral arteries very early after induction of SAH. In addition, ERK1/2 activity at 24 h after SAH is effectively inhibited by U0126 treatment when given at 6 h after SAH, which is the starting time of the U0126 treatment in both treatment regimens A and B. 

### Effect of U0126 treatment on SAH induced upregulation of cytokines and MMP-9

**Figure 7:**
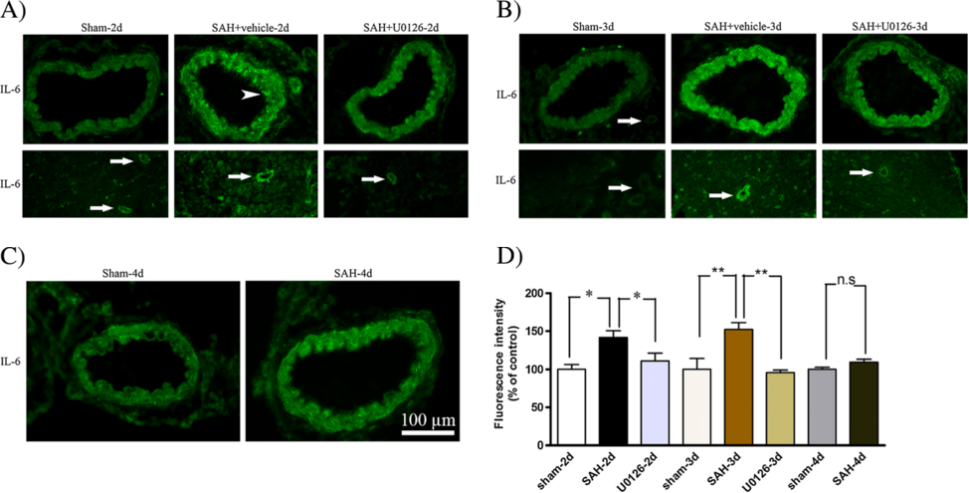
**Interleukin (IL)-6 immunoreactivity in the walls of the middle cerebral artery (MCA) and microvessels following subarachnoid hemorrhage (SAH). **Images represent (**A**) sham, SAH + vehicle and SAH + U0126, 2 days after SAH; (**B**) sham, SAH + vehicle and SAH + U0126, 3 days after SAH; and (**C**) sham and SAH, 4 days after SAH. There was a significant increase in IL-6 protein levels in the wall of the MCA and surrounding microvessels (white arrows) after SAH + vehicle as compared to sham-operated rats at 2 and 3 days with maximum at 3 days. IL-6 immunoreactivity was slightly increased in MCA at 4 days in the SAH group as compared to the sham group. Treatment with U0126 given at 6 h after induced SAH prevented the increased protein expression in both MCA and microvessels at 2 and 3 days. IL-6 was located in both SMCs and in the endothelium (arrowhead). (**D**) Bar graph showing semiquantification of fluorescence intensity for IL-6 in MCA. Data are presented as the mean percentage relative to sham ± SEM; n = 5. *P <0.05, **P <0.01.

**Figure 8:**
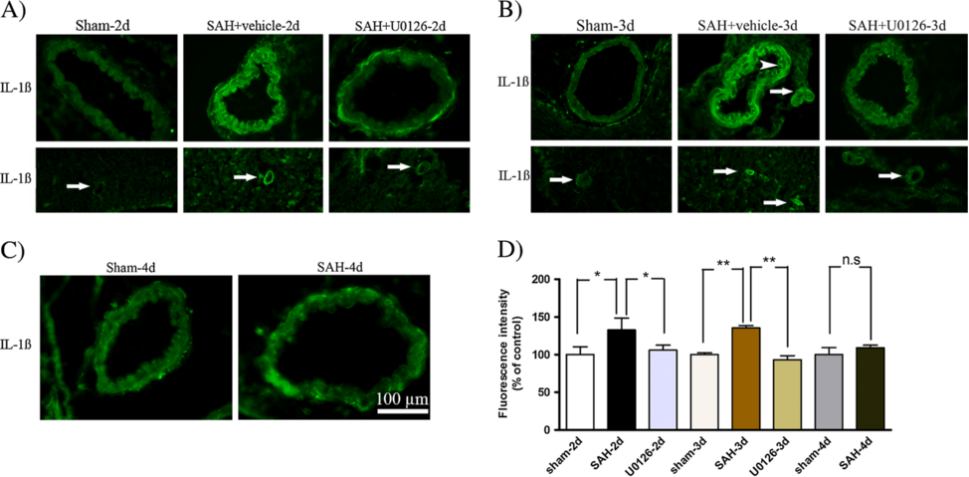
**Interleukin (IL)-1β immunoreactivity in the walls of the middle cerebral artery (MCA) and microvessels following subarachnoid hemorrhage (SAH). **Images represent (**A**) sham, SAH + vehicle and SAH + U0126, 2 days after SAH; (**B**) sham, SAH + vehicle and SAH + U0126, 3 days after SAH; and (**C**) sham and SAH, 4 days after SAH. There was a significant increase in IL-1β protein which was located in the SMCs and endothelium (arrowhead) in the MCA and in the surrounding microvessels (white arrows) after SAH + vehicle as compared to sham-operated rats at 2 and 3 days with maximum at 3 days. IL-1β immunoreactivity was slightly increased at 4 days in the MCA in SAH group as compared with the sham group. Treatment with U0126, given at 6 h after induced SAH, prevented the increased protein expression at 2 and 3 days. (**D**) Bar graph showing semiquantification of fluorescence intensity for IL-1β in MCA. Data are presented as the mean percentage relative to sham ± SEM; n = 5. *P <0.05, **P <0.01.

**Figure 9:**
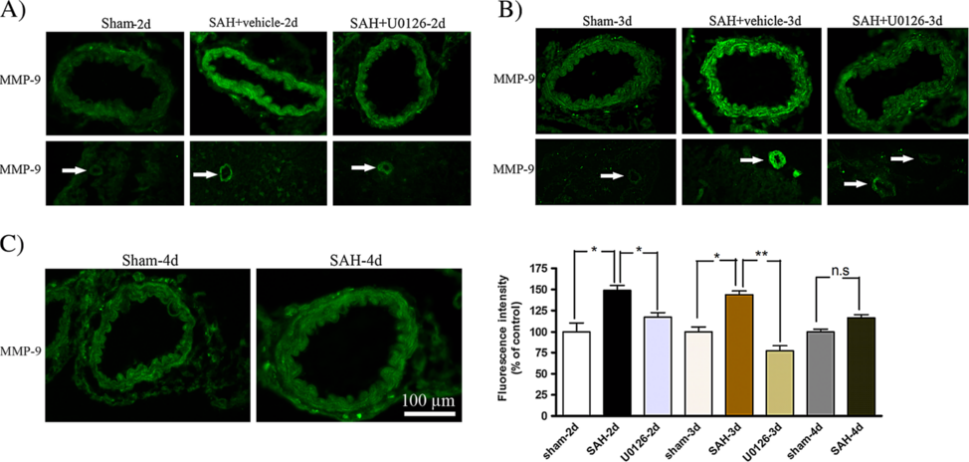
**Matrix metalloproteinase (MMP)-9 immunoreactivity in the walls of the middle cerebral artery (MCA) and microvessels following subarachnoid hemorrhage (SAH). **Images represent (**A**) sham, SAH + vehicle and SAH + U0126, 2 days after SAH; (**B**) sham, SAH + vehicle and SAH + U0126, 3 days after SAH; and (**C**) sham and SAH, 4 days after SAH. There was a significant increase in MMP-9 protein in SMCs of the MCA and surrounding microvessels (white arrows) after SAH as compared to sham-operated rats at 2 and 3 days but not at 4 days. Treatment with U0126, given at 6 h after induced SAH, prevented the increased protein expression at 2 and 3 days. (**D**) Bar graph showing semiquantification of fluorescence intensity for MMP-9 in MCA. Data are presented as the mean percentage relative to sham ± SEM; n = 5. *P <0.05, **P <0.01.

**Figure 10:**
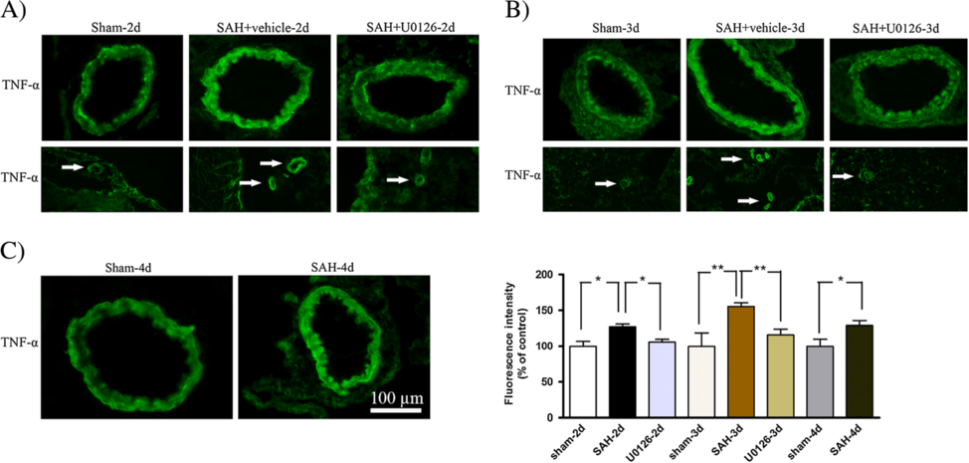
**Tumor necrosis factor (TNF)α immunoreactivity in the walls of the middle cerebral artery (MCA) and microvessels following subarachnoid hemorrhage (SAH). **Images represent (**A**) sham, SAH + vehicle and SAH + U0126, 2 days after SAH; (**B**) sham, SAH + vehicle and SAH + U0126, 3 days after SAH; and (**C**) sham and SAH, 4 days after SAH. There was a significant increase in TNFα protein level in the SMCs of the MCA after SAH + vehicle as compared to sham-operated rats at 2, 3 and 4 days with maximum at 3 days. Treatment with U0126, given at 6 h after induced SAH, prevented the increased protein expression at 2 and 3 days. In addition, treatment with U0126 decreased the TNFα immunoreactivity in the microvessels (white arrows) after induction of SAH at 2 and 3 days. (**D**) Bar graph showing semiquantification of fluorescence intensity for TNFα in MCA. Data are presented as the mean percentage relative to sham ± SEM; n = 5. *P <0.05, **P <0.01.

Localization and expression levels of IL-6, IL-1β, TNFα and MMP-9 were examined by immunofluorescence staining. As shown in Figures 7 and 8, the IL-6 and IL-1β immunoreactivities were located in the SMCs and in the endothelial cells (arrowhead) (Figures 7 and 8). These cytokines were increased significantly at 48 and 72 h after SAH in both MCA and microvessels compared to time matched sham-operated rats, whereas SAH rats terminated at 96 h post-SAH showed only slightly (non-significantly) increased levels of IL-6 and IL-1β (Figures 7 and 8). MMP-9 was primarily expressed in the SMCs of the MCA and in microvessels in the brain tissue (Figure 9), which, was significantly increased at 48 h and 72 h after SAH (Figure 9A,B), but not at 96 h post-SAH (Figure 9C) as compared to time matched sham-operated rats. TNFα was expressed in the SMCs of the MCA and in the microvessels, and this expression of TNFα was significantly increased at 48 to 96 h post-SAH compared to time matched sham-operated rats, with a maximum TNFα upregulation seen at 72 h post-SAH (Figure 10). In summary, all three cytokines studied as well as MMP-9 increased progressively in cerebral arteries until day 3 post-SAH, after which their expression declined, suggesting a transient nature of the cerebrovascular inflammatory response after SAH. 

Treatments with U0126 significantly decreased the SAH-induced upregulation of IL-6, IL-1β, TNFα and MMP-9 at 48 h post-SAH (rats treated with treatment regimen A) and at 72 h post-SAH (rats treated with regimen B) as compared to time-matched vehicle-treated SAH rats) (Figures 7, 8, 9, 10). In addition, treatment with U0126 also reduced levels of these proteins in cerebral microvessels (Figures 7, 8, 9, 10). These results demonstrate a crucial involvement of the MEK-ERK1/2 pathway in upregulation of cerebrovascular cytokines and MMP-9 expression during the first 3 days post-SAH. The finding that treatment regimen B was as effective as treatment regimen A in prevention of the cerebrovascular inflammatory response, points to the time window from 6 h to 24 h post-SAH as the critical time during which ERK1/2 activation in the cerebral arteries triggers the later (up to 3 days post-SAH) upregulation of proinflammatory cytokines and MMP-9.

## Discussion

This study is the first to investigate the timecourse of the inflammatory response, specifically changes in the expression of IL-6, IL-1β, TNFα and MMP-9, in the walls of cerebral arteries over the first 4 days following experimental SAH. Furthermore, it is the first to investigate the role of MEK-ERK1/2 activation during the first 24 h post-SAH as an early trigger of the more delayed inflammatory response in the cerebral vasculature.

The study reveals an early transient peak at 6 h post-SAH in IL-6, IL-1β and MMP-9 protein expression in cerebral artery walls followed by a stronger and more persistent increase in cerebrovascular protein expression levels of TNFα, IL-6, IL-1β and MMP-9 at 24 h post-SAH and onwards until 4 days post-SAH where a beginning decline in these markers was observed. Analysis of ERK1/2 phosphorylation in cerebral arteries revealed that at 1 h post-SAH it was already strongly activated, which is in accordance with an earlier western blot study [38]. The SAH-induced ERK1/2 activation persisted until 3 days post-SAH and thereafter declined at 4 days post-SAH. Thus, induction of ERK1/2 activity is a very early event in cerebral arteries preceding the elevation of cytokines and MMP-9 commencing somewhat later in the SMCs and peaking at 3 days post-SAH. These events are primarily concentrated to the cerebrovascular SMCs not only in the large circle of Willis arteries but also in parenchymal microvessels. This provides the first direct evidence of an associated vascular mechanism, involving both large cerebral arteries and brain microvessels.

Late cerebral ischemia in man typically occurs 4 to 12 days after SAH and involves several pathophysiological mechanisms, including cerebral vasoconstriction [39], endothelial dysfunction [40,41], blood-brain barrier breakdown [42] as well as a marked inflammatory response [2,15]. Analysis of genes involved in the events associated with SAH show a spectrum of functional systems such as those of fibrinolysis, inflammation, vascular reactivity and neuronal repair [43]. We have earlier demonstrated that the expression of several vasoconstrictor receptors is increased in cerebral arteries after SAH, and that this upregulation occurs via early MEK-ERK1/2 activation, similarly to what we demonstrate in the present study for the upregulation of proinflammatory cytokines and MMP-9. We hypothesize that the ERK1/2-mediated inflammatory response and vasoconstrictor receptor upregulation works in concert [39] to disturb the normal cerebrovascular reactivity and thereby contribute to development of delayed cerebral ischemia [44].

A large number of earlier studies have investigated the expression of proinflammatory cytokines in ischemic brain tissue where they contribute to elevated infarct size and more neurological deficits. The proinflammatory cytokines TNFα, IL-6 and IL-1β have been found in the brain infarct region [45] and the same cytokines have been suggested to be involved in the development of late cerebral ischemia after SAH [6,15]. The pattern of cytokine expression differs depending on stroke type and localization. Comparison of two SAH models revealed that the expression of TNFα, IL-6 and IL-1β were differentially increased in the brain at 2 and 7 days post-SAH [14]. Thus, following perforation of cerebral arteries the response was small and late (at 7 days), while after blood injection into basal cisterns, an increase of cytokines was found in the brain tissue at 2 days after SAH.

In the present study, we found that at early timepoints (1 to 24 h) following SAH there was a marked expression of TNFα in the brain parenchyma. Interestingly, the other two cytokines studied, IL-6 and IL-1β, did not show any enhanced expression in the brain tissue. The elevated brain TNFα expression was colocalized with GFAP, a marker of glial cells and astrocytes around the vessels and in the brain tissue. This is in accordance with the previous observations of TNFα mRNA and protein expression at an initial peak around 1 to 3 h and a second peak at 12 to 24 h in cortical neurons after focal cerebral ischemia [34], and in microglia and astrocytes after transient forebrain ischemia in rats [46]. Activation of microglia and astrocytes leading to release of cytotoxic substances such as nitric oxide and TNFα have been reported in response to early brain injury after cerebral ischemia [47]. In addition, increased production of TNFα in brain tissue has been reported following cerebral ischemia [48]. A recent study reported that blockade of endogenous TNFα may significantly reduce infarct size in rats following permanent and transient focal cerebral ischemia, suggesting involvement of TNFα in ischemic neuronal damage [49]. We speculate that reactive gliosis early after SAH may give rise to the increased brain TNFα levels observed in the present study, which may in turn play a role in early neuronal damage after SAH.

With regard to the expression of cytokines in the walls of the cerebral vasculature, we demonstrate a significantly increased expression of TNFα in the SMCs of the MCA at 2 to 4 days post-SAH. There was moderately increased TNFα expression at 24 h post-SAH, which increased slightly over time during 48 to 96 h post-SAH. This is in agreement with a previous study that showed that TNFα mRNA was elevated at 24 to 48 h in the walls of cerebral arteries after SAH [50]. In addition, another group of investigators reported on TNFα expression in the wall of the BA at 2 to 5 days post-SAH in mice [51]. Interestingly, there was no change in TNFα expression at early timepoints (0 to 12 h post-SAH) in the cerebral artery walls, indicating a shift in the SAH-induced TNFα expression from brain tissue at early timepoints to the cerebral vasculature at delayed timepoints.

For IL-6 and IL-1β, we observed a minor early increase in expression of these cytokines in the walls of cerebral arteries and microvessels at the early timepoint 6 h post-SAH, which further increased over time to reach a maximum peak at 72 h post-SAH. The findings are consistent with other investigations, which have demonstrated that IL-6 and IL-1β mRNAs and proteins were increased in cerebral vessels following transient global ischemia [52] and SAH [50].

We did not find any enhanced expression of TNFα, IL-6 and IL-1β proteins in fresh rats, 0 h group or sham operated animals. This indicates that the production and secretion of the studied cytokines correlate with the events occurring after SAH.

Cytokines are pleiotropic low molecular weight proteins with multiple diverse biological activities. The production of cytokines after SAH results in the induction of cyclo-oxygenase 2 (COX2), which is involved in the breakdown of arachnoid acid and the activation of the lipoxygenase pathway. COX2 activation after SAH has been suggested to cause cerebral artery vasoconstriction, activation and infiltration of leukocytes and neutrophils, increased vascular permeability, and increase in reactive oxygen species [53]. Moreover, there is evidence that TNFα activates SMC NADPH oxidase, which again leads to generation of reactive oxygen species, resulting in cerebrovascular constriction and reduced blood flow [54]. Accordingly, TNFα, IL-6 and IL-1β have been shown to correlate to the severity of SAH, cerebral vasospasm, development of late cerebral ischemia and secondary brain damage in primate [55].

MMP-9 is a member of the matrix metalloproteinase family of proteinases, which play important roles in remodeling of extracellular matrix components (collagen, laminin and elastin) in the walls of blood vessels [56]. It has been reported that MMP-9 following cerebral ischemia is able to degrade the endothelial basal lamina and thereby increase the permeability of the BBB [57]. Increased expression of MMP-9 has been observed in cerebral aneurysm walls in humans [58]. Previously, we reported that MMP-9 mRNA and protein levels were increased in the SMC of cerebral arteries and microvessels at 24 and 48 h after SAH [29] and focal cerebral ischemia [59,60]. In the present study we revealed an early slight increase in MMP-9 expression in the walls of cerebral arteries at 6 h and it increased to 72 h. This is in agreement with the demonstration of transcriptional MMP-9 mRNA upregulation *in vivo*[59]. However, there was very weak expression of MMP-9 in the brain tissue at all timepoints, which is in agreement with previous work [61]. We therefore speculate that the upregulation of MMP-9 is a response to SAH specific for the cerebral vasculature, and that the upregulated MMP-9 may play a role in the complex vasculopathy after SAH.

Several studies have hypothesized on the involvement of the MEK-ERK1/2 pathway in development of cerebral vasospasm after experimental SAH [62,63]. We observed a significant increase in ERK1/2 phosphorylation in the wall of the MCA following SAH. Interestingly, this expression started already at the early timepoint 1 h and remained elevated over time until 4 days post-SAH. Ansar and Edvinsson reported that only the MEK-ERK1/2 pathway was activated at early timepoints after SAH, while p38 or c-Jun N-terminal kinase (JNK) were activated only at 48 h after SAH [38]. Furthermore, Larsen and coworkers [33] reported that the specific MEK1/2 inhibitor U0126 prevented SAH-induced late cerebral ischemia in rats. Blockade of the upstream activator of MEK1/2, with a specific Raf inhibitor (SB386023-b), also prevented the enhanced cytokines and MMP-9 expression after SAH, and the associated delayed reduction in CBF [29]. We found that U0126 significantly aborted the phosphorylation of ERK1/2, which in turn decreased the enhanced expression of IL-6, IL-1β, TNFα and MMP-9 proteins in cerebral arteries and microvessels. Secondly, we tested if our hypothesis that this is due to an early ‘switch-on’ mechanisms and that treatment for only the first day would be enough. Interestingly, treatment with U0126 showed similar effects irrespectively of whether (A) we administered it at 6, 12, 24, 36 h and terminated the experiment at 48 h after SAH or if (B) we administered it at 6, 12 and 24 h and terminated the experiment at 72 h after SAH. This shows that it is irrelevant to the effect of U0126 whether the animals are left untreated from 24 h to 72 h post-SAH as long as they are properly treated in the critical period at 6 to 24 h post-SAH, thereby aborting the early ERK1/2 activation. Moreover, the fact that this and earlier studies have consistently shown that the MEK-ERK1/2 inhibitors do not have to be administered immediately after the SAH to exert their beneficial effects, but can be delayed until 6 h post-SAH, suggest a clinically feasible therapeutic time window for these treatments.

Another important observation was that the inhibition of the ERK1/2 signaling by U0126 improved animal behavior scores, including locomotor function and coordination, and spontaneous activity. This is in contrast to the recent Clazosentan studies that were positive in reducing vessels diameter but failed to improve neurological outcome [64,65]. Thus, inhibition of early MEK-ERK1/2 signaling after SAH provides a novel therapeutic target that can be treated within a clinically relevant time window and has the potential of improving outcome after SAH.

## Conclusions

The present results show that SAH induces activation of the MEK-ERK1/2 pathway at an early timepoint after SAH in SMCs of the cerebral arteries. In addition, SAH induces a somewhat delayed expressional upregulation of proinflammatory cytokines and MMP-9 in the walls of cerebral vessels and worsening of neurological scores. Inhibition of the MEK-ERK1/2 signaling pathway by a specific MEK1/2 inhibitor as late as 6 h after induction of SAH prevented the upregulation of proinflammatory mediators and improved neurological functions. Therefore, inhibition of the MEK-ERK1/2 pathway indirectly targets other transcriptional mechanisms activated following SAH and prevents some of the deleterious events induced in the cerebral vasculature and improves outcome following SAH.

## Abbreviations

BA: Basilar artery; CSF: Cerebrospinal fluid; CBF: Cerebral blood flow; DMSO: Dimethylsulfoxide; EDTA: Ethylenediaminetetra-acetic acid; ERK: Extracellular signal-regulated kinase; GFAP: Glial fibrillary acidic protein; H&E: Hematoxylin and eosin; IL-6: Interlukin-6; IL-1β: Interlukin-1beta; ICP: Intracranial pressure; JNK: c-Jun N-terminal kinase; MEK: Mitogen-activated protein kinase kinase; MMP-9: Matrix metalloproteinase-9; MABP: Mean arterial blood pressure; MCA: Middle cerebral artery; PVDF: Polyvinylidene difluoride; RIPA: Radioimmunoprecipitation assay; SAH: Subarachnoid hemorrhage; SEM: Standard error of the mean; SMCs: Smooth muscle cells; TNFα: Tumor necrosis factor α.

## Competing interests

The authors declare that they have no competing interests.

## Authors’ contributions

AM carried the immunohistochemistry experiments, participated in the design of study, statistical analysis and writing of the manuscript. GP performed *in vivo* experimental SAH model, neurological function, western blot experiments, statistical analysis and writing of the manuscript. LE conceived the study, directed the work and writing of the manuscript. All authors have read and approved the final manuscript.
